# Perioperative care of nipple-areola complex-sparing mastectomy and one-stage breast reconstruction via endoscopic axillary approach for ductal carcinoma in situ: A case report

**DOI:** 10.1097/MD.0000000000036173

**Published:** 2023-12-15

**Authors:** Li-Xia Zhang, Li Zhang, Li-Li Jiang, Hui MI, Dong Lingling

**Affiliations:** a Department of Breast Surgery, Weihai Municipal Hospital, Cheeloo College of Medicine, Shandong University, Weihai, China; b Nursing Department, Weihai Municipal Hospital, Cheeloo College of Medicine, Shandong University, Weihai, China.

**Keywords:** breast cancer, breast reconstruction, case report, microrotatotomy, perioperative management

## Abstract

**Rationale:**

Breast cancer represents a prevalent malignancy that primarily impacts women, with pronounced consequences on their overarching health. The major therapeutic approach, encompassing surgical procedures, can often culminate in mastectomy, potentially inciting psychological turmoil and disorders.

**Patient concerns:**

A patient was admitted to our facility on May 5, 2023, precipitated by the discovery of bilateral breast masses during a routine physical examination conducted 3 days before admission.

**Diagnosis:**

The breasts were symmetric, with the right nipple inverted and a palpable mass in the upper outer quadrant of the right breast, measuring approximately 5 cm × 4 cm. The mass was firm with indistinct borders, relatively regular morphology, poor mobility, and no tenderness. Outpatient color Doppler ultrasound revealed heterogeneous echogenicity in the right breast, classified as Breast Imaging Reporting and Data System (BI-RADS) category 0, along with multiple ductal dilatations. The left breast exhibited a hypoechoic area (BI-RADS 3), indicative of proliferative changes. Radiographic mammography confirmed diffuse changes in the right breast (BI-RADS 0) and proliferative signs in the left breast (BI-RADS 2). Biopsy results reveal significant atypical ductal hyperplasia consistent with intermediate-grade ductal carcinoma in situ. This patient was diagnosed as ductal carcinoma in situ of the right breast (cTisN0M0 and Stage 0), accompanied by a left breast mass.

**Interventions:**

On May 15, 2023, the patient was readmitted for further surgical intervention. Following relevant auxiliary examinations, the patient underwent nipple-areola complex-sparing radical mastectomy for the right breast, sentinel lymph node biopsy in the right axillary area, prosthesis-based breast reconstruction for the right breast, and microrotatotomy of the left breast mass on the left side on May 17.

**Outcomes:**

The patient made a successful recovery under scrupulous perioperative supervision and was discharged 7 days post-surgery.

**Lessons:**

The axillary approach for endoscopic mammary gland excision and immediate implant reconstruction permits patients to preserve the esthetics of the female form while undergoing conventional medical treatment. This methodology considerably enhances the psychophysical health of the patients, thereby marking it as an advantageous practice worthy of broad dissemination in the medical community.

## 1. Introduction

Ductal carcinoma in situ (DCIS), which accounts for up to 25% of all screen-detected abnormalities, comprises a spectrum of abnormal cell types confined to the breast ducts with variable natural history and risk for progression and recurrence.^[1]^ DCIS represents a precancerous lesion with diverse clinical manifestations and substantial heterogeneity in biological behavior.^[2]^ The absolute mortality rate associated with DCIS itself is exceedingly low. Adequately treated DCIS has a favorable prognosis, with a 20-year breast cancer-specific mortality rate of 3.3% (95% CI 3.0–3.6).^[3]^ However, approximately 14% to 53% of untreated cases progress to invasive ductal carcinoma (IDC).^[2]^ Definitive diagnosis can only be achieved through needle biopsy or excisional biopsy. In this case, the patient exhibited masses on ultrasound, accompanied by a palpable breast lump on the right and nipple retraction during physical examination. The diagnosis of right-sided DCIS was confirmed through biopsy. Surgical intervention has traditionally been a cornerstone in the treatment of DCIS.^[4]^ For the vast majority of cases, total mastectomy has been reported as a curative approach.^[5]^ Given the patient’s high esthetic demands for breast appearance, a multidisciplinary team consultation and thorough preoperative communication were conducted. By employing cutting-edge techniques, a one-stage immediate breast reconstruction via an endoscopic axillary approach and microrotatotomy of the left breast mass were performed. The patient was discharged after a 7-day recovery period. Postoperative histopathological examination confirmed the diagnosis of nonspecific invasive breast carcinoma (IDC).

## 2. Case report

**Figure 1.**
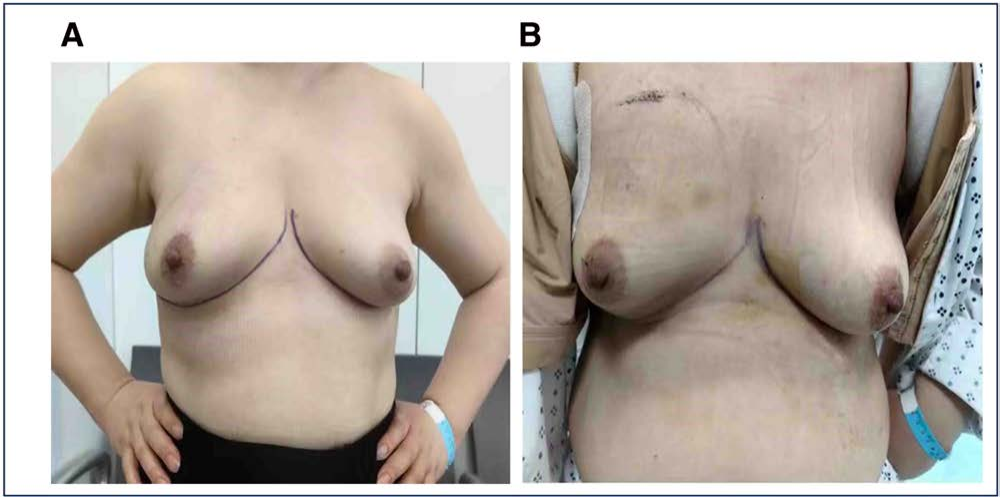
(A) Preoperative frontal position of the patient. (B) Postoperative frontal position of the patient.

A 35-year-old Chinese female patient was admitted to the hospital on May 5, 2023, due to the discovery of bilateral breast masses during a physical examination 3 days prior. Upon admission, she underwent another physical examination (Fig. 1A). The breasts were symmetrical, with no appearance of orange peel texture or other skin changes. An inverted nipple was observed on the right side, while the left nipple appeared untilted and fixed. There were no ulcerations or discharge from either nipple. A palpable mass, approximately 5 cm × 4 cm in size, was identified in the upper outer quadrant of the right breast. The mass was firm with poorly defined borders. It had a somewhat regular shape but exhibited limited mobility and no tenderness. No enlarged lymph nodes were palpated in the bilateral axillary or supraclavicular regions, and no nodules or masses were detected in the contralateral breast.

**Figure 2.**
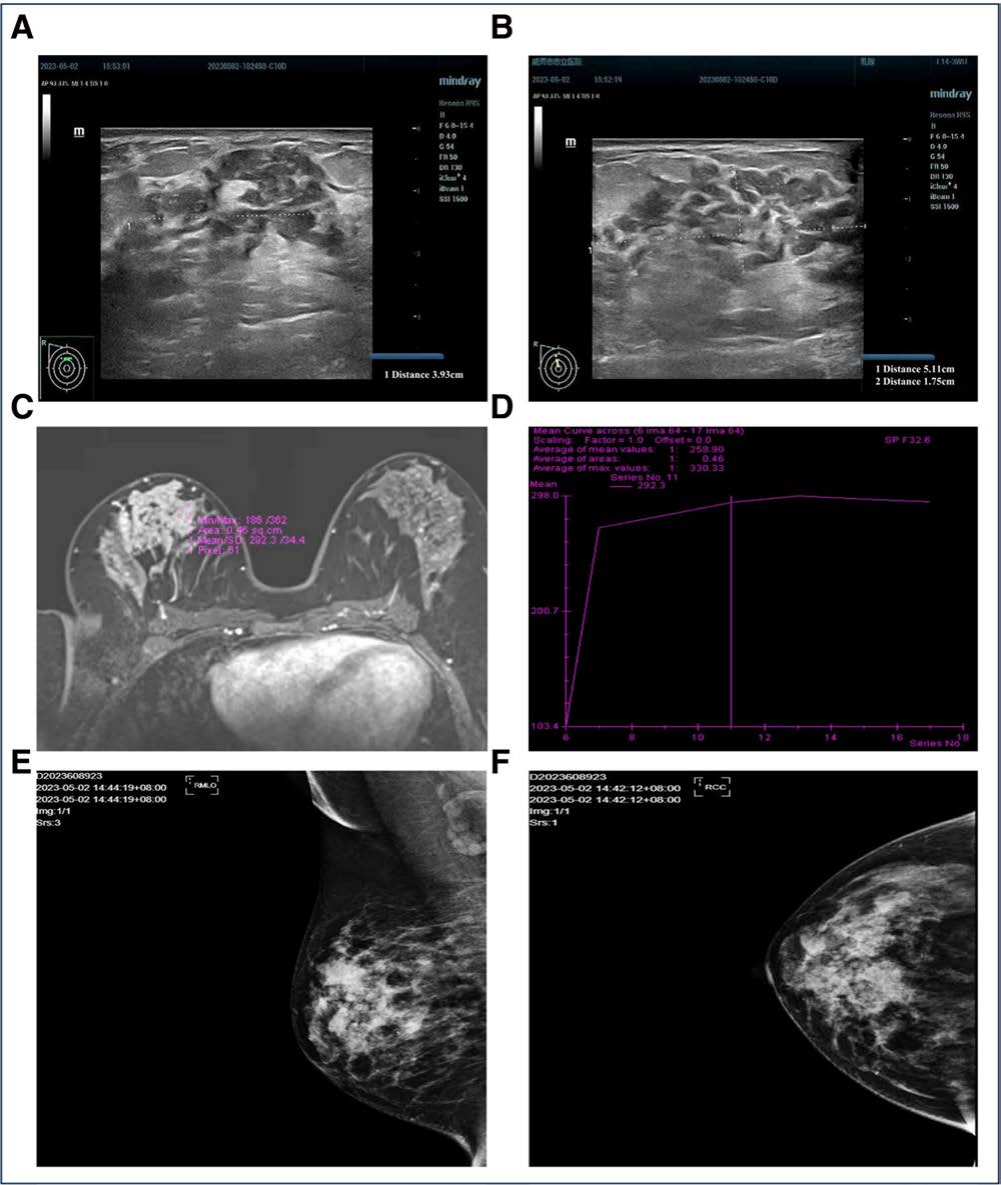
Imaging of breasts. (A and B) Color Doppler ultrasound of the right breast. (C) Radiographic mammography of bilateral breasts. Purple annotated area indicates the lesion in the right breast. (D) Breast magnetic resonance dynamic enhanced curve of purple annotated area in (C). (E) Medio-lateral oblique and (F) cranio-caudal molybdenum target X-ray image of right breast.

Color Doppler ultrasound of the breasts (Fig. 2A and B) conducted in the outpatient department demonstrated heterogeneous echogenicity in the right breast, classified as Breast Imaging Reporting and Data System (BI-RADS) category 0, indicating multiple duct dilations. The left breast showed a hypoechoic area, classified as BI-RADS category 3, suggesting proliferative changes. Additionally, magnetic resonance imaging (Fig. 2C and D) confirmed diffuse alterations in the right breast (BI-RADS category 0) and proliferative signs in the left breast (BI-RADS 2). Subsequent radiographic mammography (Fig. 2E and F) revealed abnormal enhancement and duct dilation in the upper quadrant of the right breast, indicative of a segmental distribution, classified as BI-RADS category 4B. Proliferative changes in both breasts and a small lymph node in the right axilla were also visualized.

Further examinations were conducted after admission. Results of routine blood tests and the electrocardiogram were normal, with no significant abnormalities. On the same day, under local anesthesia and ultrasound guidance, a core needle biopsy was performed on the mass in the right breast. The patient was discharged while awaiting the pathological results. On May 7, the pathology report revealed significant atypical ductal hyperplasia in the right breast mass, consistent with intermediate-grade DCIS. Immunohistochemistry results indicated: estrogen receptor (+++, 90%); progesterone receptor (+/++, 90%); human epidermal growth factor receptor 2 (++); Ki-67 (+, 10%); Calponin (basal cells +); smooth muscle myosin heavy chain (basal cells +); P63 (basal cells +); CK-H (partially +); CK5/6 (−); E-Cadherin (+). Based on these findings, the patient was diagnosed with DCIS of the right breast (cTisN0M0 and Stage 0), accompanied by a left breast mass. The clinical staging indicated that the disease was confined to the localized area [cTis], with no evidence of regional lymph node involvement [N0] or distant metastasis [M0]. Subsequently, the patient was informed of the pathology results via telephone and advised to proceed with further surgical treatment as soon as possible.

On May 15, 2023, the patient was readmitted for further surgical intervention. On admission, the patient exhibited a stable mental state, regular sleep patterns, normal appetite, bowel movements, and urination, and an average physical condition, with no significant weight changes. She reported no notable medical history or family history.

Following relevant auxiliary examinations, an MDT consultation, as well as comprehensive preoperative communication, were carried out prior to the surgery. On May 17, a minimally invasive procedure was performed under laparoscopy, which involved nipple-areola complex (NAC)-sparing radical mastectomy for the right breast, sentinel lymph node biopsy in the right axillary area, prosthesis-based breast reconstruction for the right breast, and microrotatotomy of the left breast mass on the left side. Intraoperative rapid pathological examination indicated no metastatic cancer in the sentinel lymph nodes (0/1, 0/1, 0/1, 0/1 for the respective regions) and revealed breast adenosis in 2 points on the left breast.

Postoperative pathological examination of the specimen from radical mastectomy for the right breast (without nipple, areola, and skin) showed a tumor size of 7 cm × 5.7 cm × 3.6 cm. It was histologically classified as nonspecific invasive breast carcinoma (IDC) of Grade 3. The tumor exhibited multifocal distribution, with the largest lesion measuring 0.8 cm × 0.7 cm × 0.7 cm and the smallest lesion measuring 0.3 cm × 0.3 cm × 0.3 cm in size. Intermediate-grade DCIS (intermediate nuclear grade, partly displaying comedo necrosis), approximately 6.7 cm × 5.5 cm × 3.6 cm in size, was diagnosed. Negative margins were observed at the posterior edge of the nipple-areola complex, skin edge, and deep fascia, with no intravascular cancer emboli or neural invasion. Although intralymphatic tumor emboli were present in the capsule of the sentinel lymph nodes (Stage Ia Ib), no cancer metastasis was found within the lymph nodes; there was no evidence of metastatic cancer in the sentinel lymph nodes for stages II, III, and IV (0/1, 0/1, 0/1 respectively). Immunohistochemistry results indicated estrogen receptor (1+, 85%), progesterone receptor (3+, 95%), AR (1+, 85%), C-erbB-2 (2+), wild-type P53, Ki-67 (+20%), p120 (membranous staining), E-cadherin (+), S-100 (−), D2-40 (−), CD31 (−), GATA-3 (+). In addition, negative expression of human epidermal growth factor receptor 2 was observed by fluorescence in situ hybridization.

With meticulous perioperative management, the patient was discharged 7 days after the surgery. Since she still had a drainage tube, detailed instructions were provided on how to perform home care for the drainage tube, including recording and observing the drainage volume to prevent infection. Finally, the drainage tube was removed 5 days after discharge in the outpatient department (Fig. 1B).

Post-surgery follow-up was conducted by doctors and nurses through various means such as telephone and WeChat. Currently, the patient was in good spirits, with good mobility and normal bowel movements. Besides, she has actively carried out functional exercises. Wound healing was ideal, without swelling or restricted movement in the affected limb. By wearing appropriately sized professional shaping bras and using a band for prosthesis fixation, both breasts appear symmetrical, and the patient and her husband were satisfied with the external appearance of the breasts.

Based on the Chinese Society of Clinical Oncology Breast Cancer (CSCO BC) guidelines, the patient was administered adjuvant chemotherapy (four cycles of taxotere and cyclophosphamide [TC]). As of late July, 2 rounds of adjuvant chemotherapy have been performed for the patient, experiencing only mild adverse effects.

## 3. Nursing interventions

### 3.1. Assisting patients in accepting the treatments

The patient, being relatively young, struggled to accept the diagnosis of breast cancer. Upon readmission, she faced difficulty in accepting and facing reality. Since stepping into the hospital corridor, she continuously cried on her husband’s shoulder. The nurse embraced her and gently patted her back. After comforting both the patient and family members, the nurse guided them to take a walk in the hospital park until their emotions stabilized. The approach of narrative nursing was employed to comfort the patient through this process.

In fact, the primary physician had communicated with the patient prior to her readmission to ensure that both the patient and family were aware of the necessity for surgical treatment. However, as the day for surgery approached, accepting the impending reality became increasingly challenging for the patient, leading to fluctuating moods. Following personalized communication and assessment, it was found that the patient, being a middle school teacher, had specific esthetic expectations for breast appearance. Consequently, a modified radical mastectomy and immediate breast reconstruction were introduced to the patient. Additionally, the current state of breast reconstruction within the department, as well as images of reconstructed breasts, were shown to the patient, providing her with a preliminary understanding of the surgical options. After multidimensional and comprehensive explanations and discussions, the patient finally decided to accept the one-stage breast reconstruction via an endoscopic axillary approach.

### 3.2. Prevention of infection

Postoperative incision infection is a significant cause of failure in patients undergoing breast reconstruction with the implantation of flaps and prostheses.^[6]^ Both the flap and the prosthesis are alloplastic implants that require a longer time to integrate with tissues, resulting in a longer duration of drainage tube placement compared to simple implantation procedures.^[7]^ Frequent opening of the drain bottle increases the risk of retrograde infections. The exogenous sources of infection mainly include the drainage tube, the neck of the drain bottle, and nurses’ hands. Healthcare providers should ensure adequate fluid drainage and strictly adhere to hand hygiene and aseptic techniques to minimize the risk of infection. Urinary catheterization was discontinued within 12 hours postoperatively to prevent catheter-associated infections. Prophylactic antibiotic use was initiated 30 minutes before surgery and continued postoperatively. Body temperature was closely monitored, ranging between 36.9°C and 36.2°C, with the highest recorded temperature of 37.8°C on the day of surgery.

### 3.3. Management of the drainage tube and prevention of hematoma

Vital signs were continuously monitored. Once the patient regained consciousness, the head of the bed was elevated by 15° to 30° to facilitate fluid drainage and optimal shaping of the implants. Proper fixation of the drainage tube and continuous negative pressure drainage were maintained to ensure unobstructed drainage, with close observation of the quantity and nature of the drainage fluid. The daily drainage volume gradually decreased from 119 to 60 mL, with the tube in place until discharge. Detailed instructions on managing the drainage tube and recording drainage volume were provided upon discharge. Proper management of the drainage tube has been demonstrated to be crucial in preventing the formation of hematoma and infections.^[6]^

### 3.4. Guidance of postoperative position and prevention of lower limb venous thrombosis

After the surgery, the affected limb was placed in an adduction position with the functional position crosswise, and a thin soft pad was placed under the shoulder and elbow. Upon regaining consciousness, ankle pumps were performed in bed. At noon of the first post-surgery day, 3 1-minute activities were adopted, including 1 minute in a semi-recumbent position, 1 minute for dangling the legs over the edge of the bed, and 1 minute for standing beside the bed. A total of 1 hour was spent on the activities on the first day, and it was progressively increased from the second day onwards. Daily fluid intake ranged from 1500 to 2000 mL. Within 3 days after surgery, pneumatic treatment for lower limbs was administered daily to prevent the formation of lower limb venous thrombosis.

### 3.5. Dietary guidance, pain management, and functional exercises

Encouraging patients to consume high-protein, vitamin-rich, and micronutrient-dense foods is essential for expediting wound healing. It is crucial to educate patients about avoiding carcinogenic substances in their diet, such as foods containing estrogen or growth hormones like royal jelly and bullfrog. Moreover, a high-fat diet should be avoided.^[7]^ Smoking and alcohol consumption should also be strictly prohibited.

Since the pectoralis major muscle was cut during the surgical procedure, postoperative pain was evident in the patient. Therefore, it was imperative to provide timely and accurate assessments of pain levels. Education on pain management, including informing patients about how and when to express their pain responses, such as the intensity, nature, duration, and location of the pain experienced, should be performed. Creating a tranquil recovery environment, involving adjusting lighting, reducing noise, and limiting visits from friends and relatives, was also significant during the initial 2 days following surgery.^[8]^ The patient also adhered to pain relief medication administration and cooperated with pain evaluation. Listening to music or participating in psychological counseling sessions led by instructor Tang Jing were found to be beneficial in diverting patients’ attention and alleviating pain.

Functional exercises at the bedside were introduced to the patient accordingly. Starting with exercises targeting the fingers and wrists, gradually transitioning to exercises involving the elbows and shoulders could enhance blood circulation in the affected limbs and prevent limb edema. The patient was advised against activities that may lead to prosthesis displacement, including frequent changes in body position, excessive lying down, or sitting up. Within 1 month postoperatively, activities involving arm extension, abduction, and particularly chest expansion and heavy lifting should be avoided. The patient should also avoid pressure on the reconstructed breast, such as sleeping face down or wearing tight-fitting bras. During the first 3 months postoperatively, lying flat should be preferable over lying face down, on the side, or with a high pillow. Following 4 to 6 weeks after surgery, all exercises for rehabilitation should be gradually introduced, eventually allowing full restoration of upper limb activities within approximately 6 weeks.^[9]^

### 3.6. Patient-centered care

Women with DCIS report poor patient-clinician communication and long-lasting confusion and anxiety about their treatment and prognosis.^[10]^ This patient was included in our Pink Ribbon community, where she could obtain education on breast health, view videos of functional exercises, and receive positive support from fellow patients, thereby enhancing her adherence to functional exercises. We regularly disseminate health-related information within the community to ensure patients promptly reach healthcare workers when encountering issues, obtain answers to their inquiries, and benefit from the experience-sharing among peers, ultimately elevating the overall positivity within the community.

### 3.7. Observation of skin

Skin necrosis is a significant contributing factor to the failure of flap and prosthesis implantation.^[11]^ Postoperative observations include the evaluation of skin color, temperature, capillary refill, and degree of swelling in the nipple-areolar complex and the reconstructed area. Following the surgery, surgeons provided the patient with professionally fitted compression bras, adjusting the position and tightness of the straps to allow for finger insertion in order to prevent local ischemic complications and subsequent skin necrosis. Close monitoring of flap color, temperature, and capillary refill response was also essential for assessing skin health.

### 3.8. Shaping of reconstructed breasts

Following the surgery, surgeons provided the patient with a specifically designed and well-fitted brassiere, ensuring minimal pressure on the implants.^[12]^ Nurses assisted the surgeons in changing dressings under sterile conditions and observed the upper pole, nipple, and inframammary fold position on both sides of the breasts, thereby helping the patient sense the reconstructed breasts and enhancing her confidence in disease recovery. To prevent the contraction of the pectoralis major muscle from displacing the breast implants, a band for prosthesis fixation was employed above the breast, binding and securing the implants. The patient was advised to wear this band for at least 3 months.

## 4. Discussion

NAC-sparing mastectomy is now increasingly performed in patients with breast cancer without evidence of NAC invasion due to the high patient satisfaction rate and acceptable oncologic safety. This study was an innovative exploration of reverse-order endoscopic NAC-sparing mastectomy without liposuction, combined with immediate subpectoral implant-based breast reconstruction using TiLoop Bra via a single axillary incision for a patient with DCIS.^[13]^ This surgical procedure challenges traditional endoscopic breast reconstruction.

Endoscopy-assisted breast surgery, which is performed through minimal axillary and/or periareolar incisions, has been shown to be an effective alternative for resecting malignant breast tumors. It has been performed in China for over 10 years.^[14]^ Previously, liposuction-assisted endoscopic techniques were commonly used; however, they were found to be associated with longer operation duration and greater trauma, posing significant risks of flap necrosis, bleeding, and infection. Furthermore, delayed management of postoperative complications could compromise patient safety and treatment efficacy.^[15]^

In contrast, the new approach used in this study requires only one small and inconspicuous incision beneath the patient’s axilla. Additionally, the surgical field is much clearer compared to traditional methods, resulting in fewer complications.^[16]^ Moreover, the use of an electric knife for dissection eliminates the need for liposuction, thereby reducing the operation duration.^[14]^ This surgical approach has also been beneficial in clinical practice due to its advantages in optimizing surgical plans, minimizing trauma, reducing bleeding and complications, enhancing surgical safety, promoting faster recovery, improving esthetic outcomes of the reconstructed breast, and adhering to oncoplastic principles.^[17]^ It also leaves no visible traces on the breast surface when reshaping its appearance. Thus, this surgical approach provides a new perspective for the development of minimally invasive techniques in breast surgery.

Meticulous perioperative management has the potential to prevent significant delays in postoperative adjuvant therapy due to surgical complications.^[8]^ In this study, the patient underwent active rehabilitation and functional exercise after discharge, followed by standardized adjuvant therapy. No severe adverse events were observed.

A standardized decision-making strategy facilitated the early detection, diagnosis, and treatment of the patient. Effective communication between healthcare professionals and patients played a crucial role in choosing optimal treatments, enabling the development of personalized surgical plans, ultimately benefiting the patients and improving their medical experience.

## Author contributions

**Conceptualization:** Lixia Zhang, Linlin Dong.

**Data curation:** Li Zhang, Lili Jiang.

**Formal analysis:** Lili Jiang.

**Methodology:** Hui Mi.

**Software:** Hui Mi.

**Supervision:** Linlin Dong.

**Writing – original draft:** Lixia Zhang, Li Zhang, Linlin Dong.

**Writing – review & editing:** Linlin Dong.
